# The Impact of COVID-19 on the Stock Price of Socially Responsible Enterprises: An Empirical Study in Taiwan Stock Market

**DOI:** 10.3390/ijerph18041398

**Published:** 2021-02-03

**Authors:** Kuo-Jung Lee, Su-Lien Lu

**Affiliations:** 1 Department of Commerce Automation and Management, National Pingtung University, Pingtung City 90004, Taiwan; 2 International Bachelor Degree Program in Finance, National Pingtung University of Science and Technology, Pingtung 912, Taiwan; lotus-lynn@mail.npust.edu.tw

**Keywords:** corporate social responsibility, COVID-19, event study, abnormal returns

## Abstract

This study examines the impact of the COVID-19 outbreak on the Taiwan stock market and investigates whether companies with a commitment to corporate social responsibility (CSR) were less affected. This study uses a selection of companies provided by *CommonWealth* magazine to classify the listed companies in Taiwan as CSR and non-CSR companies. The event study approach is applied to examine the change in the stock prices of CSR companies after the first COVID-19 outbreak in Taiwan. The empirical results indicate that the stock prices of all companies generated significantly negative abnormal returns and negative cumulative abnormal returns after the outbreak. Compared with all companies and with non-CSR companies, CSR companies were less affected by the outbreak; their stock prices were relatively resistant to the fall and they recovered faster. In addition, the cumulative impact of the COVID-19 on the stock prices of CSR companies is smaller than that of non-CSR companies on both short- and long-term bases. However, the stock price performance of non-CSR companies was not weaker than that of CSR companies during times when the impact of the pandemic was lower or during the price recovery phase.

## 1. Introduction

Corporate social responsibility (CSR) is valued by companies and society and is considered an important issue by academics. Commitment to CSR can improve corporate image, gain investors’ trust and contribute to sustainable development of the company. Large companies and small businesses should consider social and environmental issues. Companies that exhibit social responsibility have a competitive advantage, respond to the needs of stakeholders, can retain employees, have strong customer loyalty, and even have enhanced economic performance. The latest developments in professional associations and academia have emphasized the importance of adopting CSR strategic methods.

Generally, CSR refers to the public services performed by companies (e.g., philanthropy and charitable activities); however, this view is too narrow. Companies should engage in charitable activities, undertake economic and legal obligations, and also commit to other social responsibilities. Johnson and McCord [1] stated that a responsible company must be accountable to its employees, suppliers, local communities, and even national communities. According to [2], CSR refers to the economic, legal, ethical, and organizational expectations of a society regarding company initiatives. Wood [3] defined CSR as the configuration of relevant principles, programs, and strategies adopted for the response to social expectations. CSR refers to the obligations that companies must undertake to promote and protect the well-being of society [4]. In order to participate in social responsibility, companies must not only be accountable to their shareholders, but also to their customers, employees, suppliers and investors.

With the increasing attention paid to CSR among countries and international organizations worldwide, various practices related to CSR have also been implemented in Taiwan. For example, *CommonWealth*, a leading business magazine, initiated CSR assessments in 2007. The magazine publishes special reports on social enterprises to advocate for CSR’s importance. Annually, *CommomWealth* honors outstanding companies with CSR awards [5]. In addition, the Taiwan Stock Exchange Corporation issued “Best Practice Principles for Corporate Social Responsibility for TWSE/GTSM Listed Companies” in 2010.

The effects commitment to corporate social responsibility activities vary between companies. For example, a participation in CSR may motivate customers to buy more products, increase sales, and it may positively affect business operations or performance [6,7,8]. In addition, other studies have related that CSR can increase the insurance value of the company [9,10,11,12]. When a company is involved in a negative event, investors will reduce the selling of company’s stock, which will help slow the decline in the stock price. Godfrey et al. [11] found that CSR has a risk management effect on a company’s stock price when a negative event occurs. In other words, CSR can provide insurance value by protecting the wealth of company shareholders. Kao et al. [13] revealed that Chinese companies with CSR participation had lower firm risks, especially during the 2008 financial crisis; the effect of reducing risk due to CSR participation was strong.

The COVID-19 outbreak, which emerged in late 2019, has severely affected the global economy and people lives. Moreover, it has caused severe stock price declines. Taiwan stock market weighted index fell from 12,179 points on 14 January before the outbreak to a low of 8681 points on March 19, with a maximum drop of approximately 3500 points (29%). According to the theory of behavioral finance, emergency events will affect the basic value of stocks and the psychological and behavioral responses of investors, which further affect stock prices. As a result, the COVID-19 has a significant impact on the economy, thus influencing investor sentiment and changing inventory prices [14].

Notably, few studies have explored the effect of corporate commitment to social responsibility on corporate risk [13]. According to [15], CSR is an important emerging issue in crisis management research. Despite this research potential, CSR remains under-researched in the context of crises. This study aims to provide relevant empirical findings on this issue based on the COVID-19 outbreak. By using an event study method, this paper investigates whether the stock prices of companies committed to CSR are relatively resistant to declines during the COVID-19 outbreak.

This paper’s research contribution can be presented by comparing it with the existing literature. In terms of research topics, there are few papers [14,16] that investigate the impact of COVID-19 on the stock market, while this study further examines the CSR companies’ performance in the stock market. In addition, while previous literature has examined the relationship between CSR companies and risk in the financial crisis [13,17,18,19,20], this paper uses the stock market under COVID-19 pandemic to examine the resilience of CSR companies. In particular, this study compares the CSR stock price responses under short-term (several days) and long-term (several months) periods. In terms of research methodology, the event study approach has been used to examine many topics [21,22,23]. In this paper, we use this approach to examine CSR stock prices affected by COVID-19, which is still relatively rare.

The remainder of this study is organized as follows: The following section reviews previous studies and provides a foundation for the topic of the current study. The data and research methods are presented in the subsequent section and empirical results are discussed in Section 4. The final section provides concluding remarks.

## 2. Literature Review

The emphasis on CSR has increased among government agencies, for-profit and non-profit businesses, and the public. Increased environmental awareness has also led investors to recognize the CSR actions taken by companies. CSR has received a lot of attention from companies in recent years. Some literature has found that companies committed to CSR face lower risks.

### 2.1. CSR and Firm Risks

CSR is as a civic behavior with ethical, moral and social obligations, and it provides a basis for mutually beneficial communication between an organization and the public. Because information opacity increases the risk of stock price collapse [24,25], information disclosure is an important CSR requirement for companies. Gelb and Strawser [26] argue that companies with better CSR performance have higher level of information disclosure in their reports. Similarly, Orlitzky et al. [27] and Peloza [10] suggested that CSR can reduce the firms’ risk. El Ghoul et al. [28] also indicate that firms committed to CSR can reduce investor risk and lower corporate finance costs by decreasing information asymmetry. DeFond et al. [29] finds that more information disclosure can effectively reduce the risk of stock price collapse. Furthermore, Kim et al. [18] identifies a negative correlation between CSR performance and the risk of a stock price crash by using a sample of companies listed in the MSCI ESG index. Liu et al. [19] reveals that social trust reduces the risk of stock price collapse in the Chinese market. In addition, Rim and Ferguson [30] investigate CSR practices’ impact protecting and restoring of a company’s reputation in crises. Their results show that proactive CSR is sufficient for a company to withstand damage to its reputation as a result of a crisis. Still, it cannot be harnessed as a remedial measure after crisis.

The rationale for CSR to reduce the risk of a stock price crash is investigated in the relevant literature. Chih et al. [31] and Kim et al. [32] find that companies with high CSR tend to also provide responsible financial reporting. Such companies have limited earnings management behavior, thus resulting in high earnings quality. A company with a strong commitment to social responsibility, typically has high ethical standards. When a company invests more resources in social responsibility, it is less likely to conceal bad news from investors, which also helps balance the pursuit of all stakeholders’ interest. Because such companies have a low probability of harming their stakeholders, they have a low risk of stock price collapse. In addition, information disclosure is an important condition for CSR companies. Communication related to CSR issues may offset the negative effects of a crisis [33,34,35].

Lins et al. [17] demonstrated that CSR investments can benefit a company when the company’s overall trust and the stock market price decrease. During the financial crisis of 2008 to 2009, the stock returns of companies with high CSR were 5% to 7% higher than those of companies with low CSR. Companies with high CSR also exhibited better operational performance, stronger growth and higher employee productivity during the crisis. These results indicate that CSR not only helps companies to reduce systemic and company-specific risks, but also prevents an overall loss of trust. In addition, Wu and Hu [20] demonstrate that CSR significantly affect the risk of stock price crashes in the energy sector. They reveal that employee protection, environmental protection, product quality control, consumer protection, and supply chain partnerships significantly reduced the risk of energy companies having share price collapses.

Many studies demonstrate that corporate commitment to social responsibility activities can reduce the risk of stock price crashes. However, other studies indicate a positive relationship between CSR and the risk of stock price crashes [36,37]. That is, companies with high CSR may be subjected to more severe stock market risks than companies with low CSR. Hemingway and Maclagan [38] argue that managers may commit to CSR to conceal other unethical behaviors. Petrovits [39] and Prior et al. [40] also found that corporate management may employ CSR to hide information about a company’s finances. Quan et al. [36] proposed that CSR is positively correlated with a risk of stock price collapse. Chang et al. [37] find that CSR increase the risk of stock price collapse by using a sample of Chinese companies registered on the Shanghai Stock Exchange. They argue that over-investment in CSR has led to serious agency problems in Chinese firms.

The role of CSR in reducing corporate risks may be affected by industry-specific characteristics. El Ghoul et al. [28] reveal that companies in controversial industries, such as tobacco and nuclear industries have higher corporate risks than other companies. Because such companies already face high risks, investing in CSR is unlikely to mitigate these concerns for investors; thus, risk sharing mechanisms are ineffective in such industries. The relationship between CSR and the risk of stock price crashes remains under debate in industry and academia [20]. To obtain further information and evidence on this topic, this study examines and compares CSR firms’ impact and performance affected by the stock market crash following the COVID-19 outbreak.

### 2.2. The Impact of Covid-19 on the Stock Market

The COVID-19 pandemic has affected the lives of billions of people worldwide, and it has caused the most serious global economic crisis since the economic depression of the 1930s. In April 2020, the International Monetary Fund (IMF) estimates that world GDP will fall by 3% in 2020, and the World Trade Organization (WTO) estimates that world trade will fall by 32% [14]. The economic impact of COVID-19 has exceeded those of past endogenous and extreme events. Whereas the 2008 financial crisis had a major impact on global trade and financial markets, the current COVID-19 pandemic has disrupted demand and supply. Assessing the impact of COVID-19 on the global economy and trade has become an urgent and important issue. The pandemic situation has strained the production process and profitability of many companies. Companies are facing an unprecedented situation that was difficult to foresee, and they must pivot and learn from this crisis in the future.

COVID-19-related topics are now widely studied by scholars. For example, Gatta et al. [41] apply a machine learning approach to predict the virus spread and analyze how different lock-down strategies can effectively influence the pandemic diffusion; Le et al. [42] discuss the measures that countries can take in the face of a pandemic; and Sun et al. [43] explore the impact of COVID-19 on green consumption behavior. This study focuses on the stock market. Many studies explore the impact of emergency events on the stock market, but few investigate the impact of public health events [16]. Those studies mainly focus on the impact of SARS and influenza on the stock market. For example, Goh and Law [44] show that the Asian financial crisis in 1997 had a significant impact on tourism. According to Chen et al. [45], SARS caused Taiwan’s hotel industry stock price to plummet. They also investigate the impact of the SARS outbreak on the stock markets in Asia, and they find that SARS significantly influenced financial integration. Mctier et al. [46] investigate the impact of influenza on U.S. stock markets, and they reveal that the increase in influenza rate reduced enthusiasm for trading activities and diminished stock market returns.

As a public health event with global visibility, the COVID-19 outbreak has had a significant and lasting negative impact on national economies [47]. Narayan and Phan [48] investigate the impact of the COVID-19 outbreak on stock markets and the reactions of individual governments. Sobieralski [49] examined the impact of the outbreak on the aviation industry and job market. Moreover, Lee [16] uses big data to study the initial impact of the COVID-19 outbreak on sentiment in U.S. financial markets. The results clarify the initial impact of COVID-19 outbreak on sentiment in U.S. financial markets for various industries.

## 3. Research Methods and Procedures

### 3.1. Study Sample

An event study is adopted to examine companies’ stock market performance during COVID-19 outbreak with the aim of highlighting the effect of CSR on stock price collapse. First, a distinction is established between CSR and non-CSR firms. We use the ratings provided by *CommonWealth* magazine in Taiwan to identify companies committed to CSR activities. Since 2007, the *CommonWealth* magazine has identified Taiwan’s *Top 50 Corporate Citizens* by referring to international CSR-related indicators and assessment methods [5]. These selections are based on the dimensions of company commitment, corporate governance, and social involvement. Many studies [5,50,51,52] used the data to study CSR-related topics. These data have also been used by *CommonWealth* magazine and Yuanta Securities Investment Trust in Taiwan to issue CSR indices since 2012 [14].

The study sample includes companies listed on the Taiwan Stock Exchange (TWSE), for which data is available from the Taiwan Economic Journal (TEJ) database. This paper classifies these companies as CSR companies (those that have demonstrated commitment to CSR over the past 10 years and have received CSR awards from *CommonWealth* magazine) and non-CSR companies (those that have not received CSR awards). Of the 941 companies listed on the TWSE, 86 were classified as CSR companies.

### 3.2. Event Study Method

The event study method is a common research method in the field of management [21,22,23]. This paper uses the event study to calculate abnormal returns (ARs) in the stock market and investigate the effects of the COVID-19 outbreak. When estimating ARs in the model, we must select a period of time to build the expected model, which is called the estimation period. The period that may be affected by an event is called an event period or event window. There is no objective standard for the length of the estimation period. If the estimation period is too short, the predictive power of the model will be inadequate; if the estimation period is too long, there may be structural changes during the period. Peterson [53] suggests that if daily data are used to build the forecast model, the estimation period is usually set between 100 and 300 days. If monthly observations are used, the estimation period is usually set between 24 and 30 months. The estimation period of this study model is set at 260 days for daily data and 78 weeks for weekly data.

With respect to the choice of event window, although a longer event window is more favorable for determining an event’s influence on stock prices, such a window also makes the study results more susceptible to interference from other factors. Previous studies usually included 1 day before and 1 day after (−1, 1) the event announcement as the event window [21,22,23]. Due to price limits, stock price responses in Taiwan lack efficiency. In addition, event details may be leaked before the announcement; therefore, longer event windows are usually preferred for capturing related information. This study includes the 3 trading days before and 10 trading days after (−3, 10) the date of the event announcement as the event window; during this period, we observe the effect of the COVID-19 announcements on AR variations. In addition, to further investigate the long-term impact of the outbreak on companies’ CSR, we use weekly data with an event window of (−1, 20). In this study, we used the first COVID-19 event in Taiwan as the event date (i.e., 21 January 2020) as the event date. It is important to note that there are limitations to the use of event study. If other significant events occur during the event period, they may interfere with the impact of the event on the stock market. During the research period of this paper, there may be some smaller events that affect individual companies. However, COVID-19 should be the most significant and comprehensive event during this period. To calculate the ARs, we use the market model based on the following equation:

(1) $$R_{it}=\alpha_{i}+\beta_{i}R_{mt}+\varepsilon_{it}$$

where *R_it_* is the one-day compounded return, *R_mt_* is the stock market index return on day *t*, $\alpha_{i}$ and $\beta_{i}$ are the estimated market model parameters, and $\varepsilon_{it}$ is the daily risk-adjusted residual for firm *i*. Then, the estimated return on the stock of firm *i* on day *t* is given by:

(2) $${\hat{R}}_{it}=\alpha_{i}+\beta_{i}R_{mt}$$

We then calculate the AR of firm *i* on day *t* as follows:

(3) $$AR_{it}=R_{it}-{\hat{R}}_{it}$$

Finally, we compute the cumulative abnormal returns (CARs) easily for each time interval by summing the ARs within this window:

(4) $$CAR\left(t_{1},\;t_{2}\right)=\overset{t_{2}}{\underset{t_{1}}{\sum }}AR_{it}$$

The event study method is thus used to calculate the ARs and CARs after the COVID-19 outbreak to understand and compare the long-term impacts of the outbreak on companies’ CSR. 

## 4. The Impact of COVID-19 on the Stock Price of CSR Companies

This study uses the event research method to explore the impact of the COVID-19 outbreak on the Taiwan stock market. For this process, we analyze the stock market performance of CSR companies and compare the performance between CSR and non-CSR companies. The sample data for the empirical results are obtained from the TEJ database. The performance of the Taiwan stock market after the COVID-19 outbreak is shown in Table 1. The Taiwan stock market’s weighted stock price index dropped by 4.19% after the COVID-19 outbreak in January 2020. Average stock prices declined across all industries, with the travel industry exhibited the greatest decrease of 11.30%. Overall, the stock market showed a downward trend in February, but the greatest decrease in stock price was in March. During this month, the weighted stock index decreased by 14.03%, stocks for the tourism and aviation industry decreased by 17–18%, and stocks for the automobile industry decreased 21.58%. However, the stock market regained some of these losses in April, with the weighted index increasing by 12.23% and stocks for the aviation industry increasing by 20.16%. During May, stock prices fluctuated slightly and appeared to return to normal. Stocks for the biotech and medical industry increased by an average of 8.97% following a surge in the sales of masks and pharmaceuticals. Travel industry stocks also increased by 14.6% based on a gradual recovery in domestic travel. This result shows that the performance of the stock market varies across industries after the outbreak. This also means that the COVID-19 pandemic has caused different impacts in different industries and the result is similar to that of He et al. [14].

This study examines the impact of the COVID-19 outbreak on the Taiwan stock market by using the event study method. The AR results are presented in Table 2. From the aforementioned research method, ARs represent the difference between actual returns and expected returns. If the ARs are significantly positive, it means that the event has a positive impact on the stock price; if the ARs are significantly negative, it means that the event has a negative impact on the stock price. We use the symbols *, **, and *** to denote significance at the 10%, 5%, and 1% levels, respectively. As shown in Table 2, the ARs of 1 day before the COVID-19 event were nonsignificant for the overall company, indicating no significant stock price reaction. The ARs on the day of the event and for the following 5 days were significantly negative, indicating that the COVID-19 outbreak caused a significantly decline in the overall stock price.

For CSR companies, ARs were negative but nonsignificant on the day of the event and positive but nonsignificant for the following 2 days, indicating that the COVID-19 outbreak did not cause a significant decrease in CSR companies’ share prices. This indicates that investors have more confidence in CSR companies and that their stock prices are relatively resistant to declines under a pandemic’s risk. For non-CSR companies, AR was negative but nonsignificant on the event day, however, ARs were significantly negative for the 5 days after the event. This result indicate that the pandemic affected these companies and caused a decline in their share prices. 

In line with some recent literature [14,16], this study examines the impact of the COVID-19 pandemic on the stock market. However, this study further examines the performance of CSR firms in the stock market. In this paper AR results show that CSR companies were affected by the COVID-19 but the stock price decline was not significant. The stock prices of the overall companies and non-CSR companies were significantly affected by the COVID-19.

To understand the cumulative impact of the COVID-19 on the stock market, this study also investigates the CARs of firms following the outbreak based on an event window of (−3, 10). The results were cumulated for ARs starting 3 days before the outbreak and are presented in Table 3. Table 3 shows that the results are similar for all companies and non-CSR companies. The CARs on the day of the outbreak were all significantly positive, indicating that the cumulative effect on the first day of the outbreak was still unaffected. However, on the second day, the CARs started to change to significantly negative values and continued until the tenth day. The results show a significant negative cumulative effect on the stock price on the second day of the outbreak and the effect persists until the tenth day. This indicates that the COVID-19 outbreak impacted the Taiwan stock market, causing a significant stock price decrease. For CSR companies, the CARs were positive but nonsignificant on the event day, positive for the first 2 days after the event day and negative but nonsignificant from the third day after the event. This result indicates that the COVID-19 outbreak nonsignificantly affected CSR companies. The effect was a relatively small decrease in stock prices. This result also reflects that investors had more confidence in CSR companies during the stock market downturn and that the share prices of CSR companies are relatively resistant to declines.

Although the impact of the COVID-19 pandemic has been, at the time of writing, less severe on Taiwan than on other countries, its effect on the Taiwan stock market continued for several months. In contrast to the previous literature [13,17,18,19], this study investigates the impact of the CDVID-19 on CSR companies in the short and long term. The results in Table 2 and Table 3 only present the effects for a few days after the first case of the outbreak and do not fully reflect the effects of the outbreak. In this study, the ARs and CARs were recalculated using weekly data to present the effects over a longer period of time. The event window is from 1 week before to 20 weeks after the outbreak. This time period includes the stock market recession and rebound and allows a more complete examination of the impact of COVID-19 on the Taiwan stock market. 

The results presented in Table 4, indicate that the AR for the week of the event (t = 0) was significantly negative for overall firms. During the seventh week (t = 6), the AR was −4.03, which was significantly negative. This was the week when the stock market was most affected by the pandemic. After the ninth week, the stock market stabilized and stock prices gradually recovered. From week 9 to week 21, the ARs were sometimes significantly positive and sometimes significantly negative. The positive ARs were significantly larger than the negative ones, indicating that the stock market gradually stabilized and recovered after the severe decline in weeks 7 (t = 6) and 8. The results for non-CSR companies were similar to the overall response, with share prices falling significantly after the COVID-19 outbreak and remaining low for approximately 3 weeks. The stock price declines were greatest in weeks 7 and 8. After week 9 (t = 8), the stock market gradually stabilized and recovered.

For CSR companies, the AR for the week of the event was −0.31, but this was not significant. The ARs for week 2 (t = 1) and 3 were also nonsignificantly negative. Compared to non-CSR companies, CSR companies’ average stock price still felt following the outbreak, but the extent of decline was smaller for CSR companies than for non-CSR companies. This indicates that investors trust CSR companies and their stock prices are relatively resilient to the impact of COVID-19. During the seventh week of the outbreak (t = 6), when the impact on the Taiwan stock market was greatest, the AR of CSR companies was −1.09, which was significantly negative. This shows that the share price of CSR companies also had a significant decline. In week 8 (t = 7), the AR of CSR companies was −1.03 (nonsignficant). This indicates that the downward trend in share prices slowed during week 8. Compared with non-CSR companies, the share price declines were less pronounced in CSR companies than in non-CSR companies during the week of the event. However, share price declines were less severe for CSR companies than for non-CSR companies; moreover, during the worst stock market decline, the share price of CSR companies recovered more quickly than did those of non-CSR companies.

In this study, using CARs, we explore the persistent and cumulative effects of the pandemic on the stock market. The results are presented in Table 5; CARs for the week before the COVID-19 outbreak were non-significantly positive for all companies and for non-CSR companies. This indicates that the outbreak had not affected the stock market by this week. After the outbreak, all stock prices were significantly impacted in the first week (t = 0). The impact continued until the fourth week (t = 3), when it gradually disappeared. However, the positive CAR changed to −4.02 in the seventh week (t = 6), indicating that all companies’ stock prices were affected by the COVID-19 and decrease again. The CAR then gradually decreased until the fourteenth week (t = 13). During this week, the CAR was −0.95 (significantly negative); CARs were positive in weeks 15 and 16. The results indicate that the impact of the pandemic on stock prices stabilized, after which the stock prices exhibited an upward.

By contrast, the CARs of CSR firms in the week of the outbreak (t = 0) were non-significantly negative. These results reveal that the effect of the COVID-19 outbreak was less severe on CSR firms than on non-CSR firms. The same was true for the following week (i.e., the CAR was non-significantly negative). CAR only became significantly negative in the third week (t = 2; CAR= −0.86), indicating that the impact of the COVID-19 outbreak on the stock market was delayed. Compared with companies overall, CSR companies were apparently less affected by COVID-19 and their stock prices were relatively resistant to decline. In the fourth week (t = 3 the CARs of other firms remained significantly negative, but the CARs of CSR firms became non-significantly positive. The CARs of other firms remained significantly negative from week 7 (t = 6) to week 12, whereas the CARs of CSR firms were non-significantly negative. These results show that the effect of the COVID-19 outbreak on the stock market was less severe for CSR companies than for non-CSR companies, moreover the share price declines for CSR companies were relatively smaller.

In summary, the outbreak had a significant impact on the overall stock market in Taiwan after the first case was announced and for 14 weeks following this announcement. The stock prices of non-CSR companies were severely impacted by the COVID-19 outbreak. This effect stabilized after the twelfth week (t = 11). The cumulative impact of the outbreak was less pronounced for CSR companies after week 3 (t = 2).

To compare CSR and non-CSR companies, the mean difference between the ARs and CARs of these two types are further examined. Table 6 compares the daily ARs and CARs of CSR and non-CSR firms for event windows of (−1, 5) and (−3, 10). The average ARs of CSR firms for these event windows are −0.125 and −0.024, respectively, which are higher than the average ARs of non-CSR firms (−0.305 and −0.163, respectively). This indicates the effect of the COVID-19 on CSR companies was less severe than that on non-CSR companies; however, the difference in ARs is not significant. Besides, CSR firms’ average CARs for the same event windows are 0.036 and −0.032, respectively, which are significantly higher than those of non-CSR firms (−0.522 and −0.885, respectively). This indicates that CSR firms outperformed non-CSR firms under the impact of the pandemic. 

For weekly intervals, the first three weeks, the weekly average ARs of CSR firms are non-significantly higher than those of non-CSR firms; the respective three CARs of CSR firms are −0.302, −0.317 and −0.621, which are significantly higher than those of non-CSR firms. This result shows that the cumulative decrease in stock prices following the COVID-19 outbreak was significantly less severe for CSR companies than for non-CSR companies. Moreover, this indicates that investors are more confident in CSR companies than in non-CSR companies and that the stock prices of CSR companies were relatively resistant to decreases during the COVID-19 outbreak. This result is consistent with the findings in the related literature [13,17,18,19,20,30,31,32].

Besides, this study compares CSR and non-CSR firms’ stock price performance after the stabilization of the stock market. According to Table 4 and Table 5, all companies’ recovery phases after the outbreak are defined by event windows of (8, 20) for ARs and (12, 20) for CARs. As shown in Table 6, the average AR of non-CSR firms (0.750) was higher than that of CSR firms (0.218), but the difference was not significant. This indicates that non-CSR firms’ performance was not weaker than that of CSR firms during the stock price recovery phase. The same result was also observed for CARs. For the event window of (12, 20), the CARs of non-CSR and CSR firms are 0.688 and 0.097, respectively, but difference is nonsignificant. This also indicates that the cumulative stock price returns of non-CSR were not weaker than those of CSR firms. This difference relative to the aforementioned severe stock price decline may be because that stock prices at this stage were particularly responsive to companies’ profits after the implementation of relief measures. During the outbreak and stock price collapse phase, stock prices fell for most companies, except for some biotech medical companies, due to panic and uncertainty. At this stage, CSR companies were less affected by the pandemic because investors were relatively confident in them, and their stock prices were relatively resilient. As the price decline eased and the stock market gradually recovered, investor confidence was restored and the stock price again reflected future profits. For example, after the pandemic restrictions are lifted, company’s stock price that has increased profitably will likely increase. Moreover, the impact of CSR factors was relatively low during this phase. Therefore, this phase’s results indicate that the stock market performance of non-CSR companies was not weaker than that of CSR companies.

He et al. [14] investigated the impact of COVID-19 in different industries. This paper further compares the impact of COVID-19 on CSR and non-CSR in different industries. Due to the limitation of the sample of CSR companies, we examine some industries, including electronics industry, banking and insurance, automobile, trade and general merchandise, and aviation industry. The results are shown in Table 7, which includes daily and weekly data and results for different event windows. In the electronics industry, CARs for both CSR and non-CSR companies were mostly negative, indicating that companies were hit by COVID-19 in the first 11 weeks, causing stock prices to fall. However, the CARs of CSR and non-CSR companies were 1.973% and −0.692%, respectively in the 13 weeks after the outbreak. The results show that CSR companies’ stock prices recovered significantly and outperformed non-CSR companies during this period. The CARs for CSR companies in the electronics industry are significantly larger than those for non-CSR companies. For CSR companies, the impact of the pandemic is smaller than that of non-CSR companies, which is similar to that of the overall stock market. The results for the automobile industry are similar to those for the electronics industry. The result of the trade industry is also similar to that of the electronics industry. However, the CARs (12, 20) of CSR and non-CSR companies are 3.196% and 3.945%, respectively, and the difference is significantly negative. This indicates that 3 months after the COVID-19 outbreak, non-CSR companies in the trade and general merchandise industry performed better than CSR companies in the stock market.

The results for the banking and insurance and aviation industries are different from those for the electronics and automobile industries. The CARs of non-CSR companies were significantly larger than those of CSR companies. This result reflects that non-CSR companies in these two industries were relatively less affected by the pandemic. It may be that the sample of CSR companies is not large enough (only 10 and 7 respectively). It may also be that these two industries are more unique, especially banking and insurance. These companies are subject to more government regulation and requirements, have less volatility in their stock prices, and are trusted by investors. The CARs of CSR and non-CSR companies in the banking and insurance industries are mostly positive, which means that their stock prices are less affected. This table indicates that the comparison between CSR and non-CSR companies is influenced by industry category. This result is similar to that of He et al [14]. The results in Table 6 and Table 7 indicate that CSR companies are significantly less affected by the COVID-19 than non-CSR companies in the overall Taiwan stock market. However, in the banking and insurance and aviation industries, the performance of CSR firms is not as good as that of non-CSR firms.

## 5. Conclusions

CSR is one of the most important emerging issues in crisis management research. However, few studies investigate the effect of a company’s commitment to CSR on corporate risk. The relationship between CSR and the risk of stock price crashes remain under debate in the market and academia. The present empirical study investigates this relationship through the event study method in the context of the COVID-19 outbreak.

This study examines the performance of CSR firms in the stock market during the COVID-19 outbreak. We discuss the changes observed in the Taiwan stock market following the outbreak, and we compare the impact of the outbreak between CSR and non-CSR firms. The Taiwan stock market decreased by up to 3500 points during the COVID-19 outbreak at the beginning of 2020. We explore the role of CSR in mitigating this severe drop in stock prices. According to the empirical results, the ARs for the overall stock market were significantly negative on the day of first outbreak in Taiwan (21 January 2020) and for the following 5 days. The results demonstrate that the COVID-19 event impacted the overall stock market and caused a significant decline in stock prices. According to the CARs results, CSR companies had positive and negative CARs after the event, but neither was significant. This result indicates that CSR companies were non-significantly affected by the outbreak (i.e., the outbreak caused a relatively minor decline in their stock prices). This result is consistent with the findings in the related literature [13,17,18,19,20,30,31,32].

Because the COVID-19 outbreak affected the Taiwan stock market for several months, this study also investigates the impact of the pandemic on CSR companies by using the event study method with weekly data. The most severe impact of the outbreak on the overall stock market was in the seventh week. After the ninth week, the stock market stabilized and stock prices gradually recovered. Compared with the overall stock market, CSR companies were not significantly affected by the outbreak. The decline in CSR companies’ share price slowed during the eighth week, indicating that their share prices recovered faster than those of the overall stock market. On the basis of CARs, the overall stock market in Taiwan was significantly affected by the outbreak for 14 weeks; CSR companies’ stock prices were less affected by the outbreak, and the cumulative impact of the outbreak on CSR companies was less pronounced than that on the overall stock market after the third week. This indicates that investors had more confidence in CSR companies during the stock market downturn and that CSR companies’ stock prices are relatively resistant to declines. However, this result is influenced by industry factors. In the banking and insurance and aviation industries, the performance of CSR firms is not as good as that of non-CSR firms.

The mean difference in the short (7 days) and long (12 weeks) periods shows that CSR companies were less affected by the pandemic’s cumulative impact than were non-CSR companies. This study further compares CSR and non-CSR companies’ stock price performance after the outbreak has been less severe and the stock market has stabilized. The empirical results show that the stock price performance of non-CSR firms was not weaker than that of CSR firms during the stock price recovery phase. This may be related to the fact that the stock prices at this stage were particularly responsive to profits after the COVID-19 has eased.

He et al. [14] found that the impact of COVID-19 on the stock market is different for different industries. Due to the small sample size of CSR companies, this study compares the results for only a few industries. Future research could discuss whether CSR companies have different stock market performance in more industries in the face of the impact of the COVID-19 pandemic. In addition, in the application of the event study method, the most common market model is used to estimate the expected return in this study. Future studies may consider other models such as three-factor model and GARCH model and compare the results of different models.

## Figures and Tables

**Table 1:** The impact of COVID-19 on the stock market.

Industry	Stock Returns (%)				
	January	February	March	April	May
Taiwan weighted stock	−4.185	−1.765	−14.028	13.227	−0.455
Banking and insurance	−1.715	0.395	−16.386	10.784	−0.313
Foods	−2.241	0.433	−9.222	7.815	2.593
Construction	−2.895	−4.706	−9.895	14.290	0.034
Electronics industry	−4.503	−2.121	−12.863	13.180	−1.013
Trade and general merchandise	−2.894	0.657	−4.429	10.685	−0.280
Tourism	−11.291	−3.867	−17.580	17.815	14.601
Aviation industry	−7.348	−2.176	−18.850	20.162	0.794
Biotech medical	−5.659	0.600	−15.133	15.572	8.971
Steel	−3.655	−0.3153	−15.783	6.7353	−0.329
Automobile	−7.608	−1.939	−21.580	15.746	−0.859

**Table 2:** Daily abnormal returns of companies.

Event Date	All Companies		CSR Companies		Non-CSR Companies	
	ARs	Statistics	ARs	Statistics	ARs	Statistics
−3	0.57 ***	11.66	0.34 ***	3.39	0.60 ***	11.25
−2	−0.04	−0.79	0.14 *	1.70	−0.05	−1.07
−1	0.07	1.40	0.08	0.82	0.07	1.27
0	−0.21 *	−1.64	−0.32	−1.03	−0.20	−1.41
1	−0.26 ***	−3.24	0.01	0.08	−0.28 ***	−3.31
2	−0.93 ***	−9.36	0.02	0.09	−1.03 ***	−9.62
3	−0.19 ***	−2.61	−0.59 ***	−4.02	−0.16 *	−1.94
4	−0.18 ***	−2.77	−0.02	−0.14	−0.20 ***	−2.79
5	−0.32 ***	−4.68	−0.06	−0.45	−0.34 ***	−4.71
6	−0.07	−1.11	0.23 *	1.71	−0.10	−1.44
7	−0.23 ***	−3.81	0.04	0.30	−0.26 ***	−3.99
8	0.01	0.22	−0.17	−1.44	0.03	0.50
9	−0.11 *	−1.92	−0.31 **	−2.23	−0.09	−1.46
10	−0.22 ***	−4.66	0.27 *	1.94	−0.27 ***	−5.39

*, **, and *** denote significance at the 10%, 5%, and 1% levels, respectively.

**Table 3:** Daily cumulative abnormal returns of companies.

Event Date	All Companies		CSR Companies		Non-CSR Companies	
	CARs	Statistics	CARs	Statistics	CARs	Statistics
−3	0.57 ***	11.66	0.34 ***	3.39	0.60 ***	11.25
−2	0.53 ***	7.39	0.48 ***	3.84	0.54 ***	6.88
−1	0.60 ***	6.66	0.56 ***	3.63	0.61 ***	6.18
0	0.39 **	2.25	0.24	0.70	0.41 **	2.19
1	0.13	0.65	0.25	0.65	0.13	0.57
2	−0.80 ***	−2.91	0.27	0.51	−0.90 ***	−3.02
3	−0.99 ***	−3.87	−0.32	−0.69	−1.05 ***	−3.79
4	−1.18 ***	−4.50	−0.34	−0.70	−1.25 ***	−4.43
5	−1.49 ***	−6.11	−0.40	−0.87	−1.60 ***	−6.04
6	−1.56 ***	−5.75	−0.17	−0.34	−1.70 ***	−5.76
7	−1.79 ***	−6.40	−0.13	−0.22	−1.95 ***	−6.46
8	−1.78 ***	−6.48	−0.29	−0.55	−1.92 ***	−6.47
9	−1.89 ***	−7.08	−0.60	−1.25	−2.01 ***	−6.97
10	−2.11 ***	−7.98	−0.33	−0.67	−2.28 ***	−7.99

**, and *** denote significance at the 5%, and 1% levels, respectively.

**Table 4:** Weekly abnormal returns of companies.

Event Date	All Companies		CSR Companies		Non-CSR Companies	
	ARs	Statistics	ARs	Statistics	ARs	Statistics
−1	0.07	1.32	0.08	0.82	0.06	1.19
0	−0.49 ***	−3.02	−0.31	−0.88	−0.51 ***	−2.87
1	−1.71 ***	−12.40	−0.42	−1.47	−1.84 ***	−12.40
2	−0.27 **	−2.13	−0.22	−0.90	−0.27 **	−2.02
3	1.36 ***	11.67	1.01 ***	3.10	1.39 ***	11.26
4	0.50 ***	3.92	0.31	0.93	0.53 ***	3.84
5	0.55 ***	3.97	−0.65 ***	−2.60	0.68 ***	4.48
6	−4.03 ***	−16.43	−1.09 *	−1.82	−4.31 ***	−16.52
7	−3.01 ***	−10.42	−1.03	−1.28	−3.20 ***	−10.44
8	1.71 ***	8.30	0.65	1.08	1.81 ***	8.32
9	1.69 ***	13.46	0.58	1.48	1.80 ***	13.67
10	1.66 ***	9.68	1.26 ***	2.83	1.70 ***	9.25
11	−0.71 ***	−4.31	−1.92 ***	−6.26	−0.59 ***	−3.31
12	1.84 ***	12.00	0.55 *	1.88	1.97 ***	11.92
13	−0.11	−0.70	0.47	0.98	−0.18	−1.05
14	1.57 ***	9.52	−0.11	−0.26	1.74 ***	9.94
15	−0.52 ***	−2.95	0.32	0.73	−0.59 ***	−3.17
16	1.24 ***	6.43	1.35 ***	2.93	1.23 ***	5.93
17	0.80 ***	5.05	0.30	1.09	0.85 ***	4.94
18	−0.96 ***	−6.40	0.15	0.37	−1.07 ***	−6.73
19	−1.32 ***	−7.72	−0.86 **	−2.34	−1.37 ***	−7.42
20	2.23 ***	11.92	0.09	0.28	2.45 ***	12.11

*, **, and *** denote significance at the 10%, 5%, and 1% levels, respectively.

**Table 5:** Weekly cumulative abnormal returns of companies.

Event Date	All Companies		CSR Companies		Non-CSR Companies	
	CARs	Statistics	CARs	Statistics	CARs	Statistics
−1	0.07	1.32	0.08	0.82	0.06	1.19
0	−0.43 **	−2.29	−0.23	−0.65	−0.44 **	−2.18
1	−2.13 ***	−8.49	−0.65	−1.40	−2.28 ***	−8.37
2	−2.40 ***	−9.63	−0.86*	−1.83	−2.55 ***	−9.46
3	−1.05 ***	−3.75	0.15	0.26	−1.16 ***	−3.85
4	−0.54	−1.55	0.46	0.63	−0.64 *	−1.68
5	0.01	0.03	−0.19	−0.27	0.04	0.10
6	−4.02 ***	−8.32	−1.29	−1.31	−4.27 ***	−8.20
7	−7.03 ***	−11.06	−2.32	−1.54	−7.47 ***	−10.98
8	−5.32 ***	−9.04	−1.67	−1.27	−5.66 ***	−8.95
9	−3.63 ***	−6.05	−1.09	−0.77	−3.86 ***	−6.00
10	−1.97 ***	−3.62	0.17	0.14	−2.16 ***	−3.69
11	−2.68 ***	−5.09	−1.75	−1.42	−2.75 ***	−4.86
12	−0.84	−1.43	−1.20	−0.90	−0.78	−1.23
13	−0.95 *	−1.67	−0.73	−0.64	−0.96	−1.56
14	0.62	0.99	−0.84	−0.60	0.78	1.16
15	0.10	0.16	−0.52	−0.37	0.18	0.26
16	1.34 *	1.91	0.83	0.55	1.41 *	1.86
17	2.14 ***	2.99	1.13	0.76	2.27 ***	2.92
18	1.19 *	1.71	1.28	0.91	1.19	1.59
19	−0.14	−0.19	0.42	0.28	−0.18	−0.22
20	2.10 ***	2.69	0.51	0.32	2.27 ***	2.70

*, **, and *** denote significance at the 10%, 5%, and 1% levels, respectively.

**Table 6:** Comparison of ARs and CARs between CSR and non-CSR companies.

Data Periods	(Cumulative) Abnormal Returns	CSR Companies (%)	Non-CSR Companies (%)	t-Statistics
Day periods	AR (−1, 5)	−0.125	−0.305	1.131
	AR (−3, 10)	−0.024	−0.163	1.227
	CAR (−1, 5)	0.036	−0.522	1.531 *
	CAR (−3, 10)	−0.032	−0.885	2.739 ***
Weekly interval	AR (−1, 3)	0.029	−0.232	0.451
	AR (−1, 6)	−0.161	−0.534	0.295
	AR (−1, 10)	0.014	−0.180	0.314
	AR (8, 20)	0.218	0.750	−1.217
	CAR (−1, 3)	−0.302	−1.274	1.786 *
	CAR (−1, 6)	−0.317	−1.405	1.895 **
	CAR (−1, 10)	−0.621	−2.534	2.620 ***
	CAR (12, 20)	0.097	0.688	−1.162

*, **, and *** denote significance at the 10%, 5%, and 1% levels, respectively.

**Table 7:** Comparison of CARs between CSR and non-CSR companies across industries.

Data Periods	Cumulative Abnormal Returns	CSR Companies (%)	Non-CSR Companies (%)	t-Statistics
Electronics industry		*N* = 35	*N* = 379	
Day periods	CAR (−1, 5)	−0.091	−2.029	3.535 ***
	CAR (−3, 10)	0.051	−2.040	4.670 ***
Weekly interval	CAR (−1, 3)	−0.376	−2.473	2.979 ***
	CAR (−1, 6)	−0.678	−3.064	2.944 ***
	CAR (−1, 10)	−0.431	−4.813	4.142 ***
	CAR (12, 20)	1.973	−0.692	6.772 ***
Banking and insurance		*N* = 10	*N* = 22	
Day periods	CAR (−1, 5)	1.506	2.872	−4.005 ***
	CAR (−3, 10)	1.357	2.365	−3.211 ***
Weekly interval	CAR (−1, 3)	1.540	1.913	−0.577
	CAR (−1, 6)	2.214	2.620	−0.640
	CAR (−1, 10)	1.770	2.780	−2.126 **
	CAR (12, 20)	−0.845	−1.336	1.330 *
Aviation industry		*N* = 7	*N* = 16	
Day periods	CAR (−1, 5)	−3.221	−1.475	−2.134 **
	CAR (−3, 10)	−3.304	−2.012	−1.815 **
Weekly interval	CAR (−1, 3)	−3.235	−2.119	−0.906
	CAR (−1, 6)	−3.504	−2.165	−1.640 *
	CAR (−1, 10)	−5.279	−3.176	−1.872 **
	CAR (12, 20)	−5.378	−0.627	−5.535 ***
Automobile		*N* = 4	*N* = 29	
Day periods	CAR (−1, 5)	−1.039	−3.788	2.765 ***
	CAR (−3, 10)	−0.588	−4.457	4.608 ***
Weekly interval	CAR (−1, 3)	−0.569	−4.514	2.847 ***
	CAR (−1, 6)	−0.886	−6.450	3.641 ***
	CAR (−1, 10)	−3.339	−10.048	2.962 ***
	CAR (12, 20)	−0.911	−12.462	5.261 ***
Trade and general merchandise		*N* = 3	*N* = 19	
Day periods	CAR (−1, 5)	0.521	−2.079	3.378 ***
	CAR (−3, 10)	0.244	−2.065	4.606 ***
Weekly interval	CAR (−1, 3)	0.468	−0.885	1.330
	CAR (−1, 6)	1.139	0.117	1.007
	CAR (−1, 10)	3.857	−0.065	2.699 ***
	CAR (12, 20)	3.196	3.945	−1.507 *

*, **, and *** denote significance at the 10%, 5%, and 1% levels, respectively.

## Data Availability

Not applicable.
